# Illumination-Invariant Feature Point Detection Based on Neighborhood Information

**DOI:** 10.3390/s20226630

**Published:** 2020-11-19

**Authors:** Ruiping Wang, Liangcai Zeng, Shiqian Wu, Wei Cao, Kelvin Wong

**Affiliations:** 1 Key Laboratory of Metallurgical Equipment and Control Technology, Ministry of Education, Wuhan University of Science and Technology, Wuhan 430081, China; wangruiping@wust.edu.cn (R.W.); zengliangcai@wust.edu.cn (L.Z.); 2 Hubei Key Laboratory of Mechanical Transmission and Manufacturing Engineering, Wuhan University of Science and Technology, Wuhan 430081, China; 3 Institute of Robotics and Intelligent Systems, Wuhan University of Science and Technology, Wuhan 430081, China; cw1989@sues.edu.cn; 4 School of Information Science and Engineering, Wuhan University of Science and Technology, Wuhan 430081, China; 5 School of Electrical and Electronic Engineering, The University of Adelaide, Adelaide 5005, Australia; kelvin.wong@ieee.org

**Keywords:** neighborhood information, feature point detection, illumination invariance, large-photometric-variation, computer vision

## Abstract

Feature point detection is the basis of computer vision, and the detection methods with geometric invariance and illumination invariance are the key and difficult problem in the field of feature detection. This paper proposes an illumination-invariant feature point detection method based on neighborhood information. The method can be summarized into two steps. Firstly, the feature points are divided into eight types according to the number of connected neighbors. Secondly, each type of feature points is classified again according to the position distribution of neighboring pixels. The theoretical deduction proves that the proposed method has lower computational complexity than other methods. The experimental results indicate that, when the photometric variation of the two images is very large, the feature-based detection methods are usually inferior, while the learning-based detection methods performs better. However, our method performs better than the learning-based detection method in terms of the number of feature points, the number of matching points, and the repeatability rate stability. The experimental results demonstrate that the proposed method has the best illumination robustness among state-of-the-art feature detection methods.

## 1. Introduction

Digital images consist of limited and discrete pixels obtained using digital image sensors (such as CCD or CMOS). These discrete pixels reflect energy intensity through numerical values, and the energy intensity is related to the characteristics of the captured object. Due to the existence of this relationship, the features of the captured object can be expressed by the pixels in the image. Feature detection is an abstraction of image information and a local decision-making method for each pixel whether there is a given type of feature. It is a fundamental problem in computer vision and has many practical applications, such as object detection [1], stereo matching [2], color matching [3], and motion estimation [4]. In order to response to diverse applications, many detection methods have been proposed [5,6]. Following traditional classification methods, feature detection can be divided into point, edge, and region detection. Feature point is most widely used because of its stability and uniqueness.

Feature point detection with geometric invariance and illumination invariance has always been a challenging problem. Geometric invariance includes translation, rotation, scale, and affine invariance. The illumination invariance is also called illumination robustness. The illumination robustness of the feature detector reflects the ability to extract features from low-illumination or overexposed images. In the past, this work was often used as a supplement to geometric invariance, and there were few dedicated studies as if it were not important. However, with the widespread application of computer vision, feature point detection in complex scenes (such as non-uniform illumination) has become a must. The illumination invariance becomes as important as the geometric invariance. This paper focuses on the feature point detection method of illumination robustness, proposes a novel method of illumination-robust feature point detection.

To the best of our knowledge, the early illumination-robust detection are all feature-based methods. One of the most common methods is to improve the illumination quality of the input image. For example, Faille [7] decomposes the input image into illumination components and reflection components, and then uses a high-pass filter to remove low-frequency illumination components. Gevrekci et al. [8] apply the contrast stretching function to two differently illuminated images. When the contrast center is changed, the two differently illuminated images obtain similar response images at different contrast centers. At this time, most feature detectors can obtain a better detection result. Xue and Gao [9] constructed an illumination invariant color space based on adaptive histogram equ lization and dark channel priority theory, and then used AKAZE detector to extract feature points. Adaptive histogram equalization was used to enhance texture details and balance the illumination in the image, and dark channel priority was used to further reduce the impact of illumination on feature extraction.

Another better option is to consider the illuminance robustness during the design of the feature detector. Moravec [10] proposed the earliest corner detection method. Harris and Stephens [11] used the gradient to calculate the response function, and then used the response function to determine corners. The introduction of gradients reduced the impact of illumination on the detector. Lowe [12] proposed a SIFT feature detector, and suggested using Hessian matrix instead of Harris for keypoints selection, and redefined the keypoints response function. The introduction of the Hessian matrix makes the detector robust to illumination. As an accelerated version of SIFT, SURF [13] also uses the Hessian matrix for feature selection, and the response function is improved on the basis of the Harris detector. Lee and Chen [14] proposed a method to detect feature points using histogram information. This method constructs a Hessian matrix that does not contain the second-order partial differential equation, but it contains the histogram information of the pixel neighborhood. Miao and Jiang [15] proposed a feature detector based on a nonlinear filter which is named ROLG (Rank Order Laplace of Gaussian). The ROLG is a rank order filter, and its weight is proportional to the coefficients of the LoG (Laplace of Gaussian) filter. Wu et al. [16] proposed a detection method that utilizes optimal multi-binary images to eliminate the noise and illumination effects. Considering the problem that low-contrast image structure is easily submerged by high-contrast image structure, Miao et al. [17] proposed to construct a zero-norm LoG filter. Since the response of the zero-norm LoG filter is proportional to the weighted of pixels in the local area, the filter keeps the image contrast unchanged. Furthermore, based on the zero-norm LoG filter, they developed a new feature point detector. Hong-Phuoc and Guan [18] pointed out that most hand-crafted feature detectors rely on pre-designed structures, and this pre-designed structure will be affected by uneven illumination. They proposed a feature detector to locate feature points in the image by calculating the complexity of the blocks surrounding the pixels.

Among the feature-based detection methods, Harris is considered to be the basis for the illumination robustness of the corner detectors, and the Hessian matrix is the root cause of the illumination robustness of the spot detection methods. However, Harris was based on the autocorrelation matrix introduces textured patterns and noise while detecting corners. The Hessian matrix contains second-order partial differential, and the feature detector constructed with the Hessian matrix as the response function will inevitably introduce unstable and error points around the structure [18]. Though there are also some other illumination-robust feature detection methods, these methods are not widely used due to own limitations. For example, the Wu’s method [16] must provide a reference image when extracting feature points. The method of Hong-Phuoc and Guan [18] does not work well for severely underexposed or overexposed images.

When feature-based detection methods encounter bottlenecks, deep learning have been widely used in many fields as a brand-new problem-solving idea. Naturally, learning-based methods were also introduced into feature point detection as a new attempt.

TILDE [19] introduced a learning-based method for feature point detection, and trained the regressor through supervised learning to work normally even if the illumination changes drastically. Unlike TILDE, which only performs feature detection, LIFT [20] is a novel architecture that can perform detection, orientation estimation, and description at the same time. The training process introduces the inverse training, which can minimize the influence of illumination on feature point detection. Although LIFT can extract illumination-robust feature points well, it is still a supervised learning method. Quad-Networks [21] is an unsupervised feature point detection method. It trains a neural network in an illumination-invariant manner and uses the network to sort pixels. If some pixels can achieve higher ranking under different illumination, these pixels are selected as candidate feature points. The network obtained by this training method is an illumination-robust feature detection network, which can extract illumination-robust feature points. The unsupervised learning of SuperPoint [22] is different from Quad-Networks. It proposes pre-training the feature detector on the procedurally generated polygonal synthetic geometric data set, then uses the pre-training network to extract the feature points on the real data set and use them as label data, and finally uses these data to train the network. In addition, LF-Net [23] exploit depth and relative camera pose to create a virtual target response for the network. Through this response relationship, training can be performed without hand-crafted detector, thereby performing sparse matching. D2-Net [24] addresses the problem of poor performance of traditional sparse local features under illumination changes drastically by postponing the detection. Key.Net [25] combines the hand-crafted detector and CNN filter in the shallow multi-scale framework, which reduces the number of network parameters and ensures the detection repeatibility rate. ASLFeat [26] further improves the positioning accuracy of D2-Net keypoints.

With the widespread application of learning-based methods in feature detection, some inherent disadvantages have gradually been exposed, such as poor versatility, high training costs (time and equipment), and the need for large amounts of data for learning. In addition, the uninterpretability of learning results is also a problem that must be faced. Before these problems are solved, learning-based detection methods are not suitable in many application scenarios. In view of this, feature-based detection methods are still a key research area at present and for a long time in the future. However, feature-based detectors are basically extended based on Harris, Hessian, and FAST, and these detectors themselves do not have excellent illumination robustness. Our method is a brand-new detection method, which completely bypasses the conventional design ideas of the detector and uses the location information of eight-neighborhoods for detection. Since the eight-neighborhood of the pixel itself is very close to the position of the pixel, the detailed information can be well preserved and the illumination robustness of the detection can be improved. At the same time, our method is different from Wu’s method [16]. Based on Wu’s method, we have further deepened and expanded the types of feature points from 8 types to 250 types. The expansion of types promotes the improvement of matching accuracy and matching speed. In addition, we designed a complete illumination robustness feature detection method and analyzed its matching performance. We also added experiments with different illumination intensity and illumination direction in the Section 5 (Experimental Results). The contributions of this paper are as follows:

- This paper proposes a novel feature point detection method based on the position of the neighborhood connection. At the same time, the paper also analyzed the computational complexity of the method.
- By introducing multiple-optimal image binarization method before the feature point detection, it is ensured that the proposed detection method has better illumination invariance.
- Experimental results prove that our method has significant advantages over the current state-of-the-art method in terms of the number of matching feature points and the stability of the repeatibility rate.

This paper is organized as follows. The second section introduces a multiple-optimal image binarization method. In the third section, we propose a novel feature point classification and detection method. The fourth section proposes a classification matching method based on the third section and theoretically analyzes the time consumption of different detection methods. The experimental results are given in the fifth section, and the conclusion is presented in the last section.

## 2. Illumination-Invariant Transformation

For the image with large-photometric-variation, this paper proposes a multiple-optimal image binarization method based on the related information of two images. The multiple-optimal image binarization method can further improve the feature point detection performance of the proposed method by improving the detection environment. The method assumes that the processed images are the different illumination images obtained by the same camera for the same scene. Under this premise, combined with the monotonous increment of the camera response function (CRF) [27] and the Median Threshold Bitmap (MTB) [28] order measurement method, the threshold required for binarization can be obtained. Through the multiple-optimal image binarization method, the feature point information in the image can be retained to the maximum extent, which provides guarantee for the subsequent feature point detection.

### 2.1. Monotonically Increasing of Camera Response Function

According to the monotonous increment of the CRF, a function that converts the brightness of the scene to the intensity of the image under certain exposure conditions indicates that the modification in illumination changes the intensity of the image, but maintains their relative order. Suppose we have two images $Z_{1},Z_{2}\in R^{M\times N}$ which are two images with the same scene but of different illumination. By rearranging the pixel values in ascending order of brightness, $Z_{1}^{1}$, $Z_{1}^{2}$, …, $Z_{1}^{k}$, …, $Z_{1}^{M\times N}$ and $Z_{2}^{1}$, $Z_{2}^{2}$, …, $Z_{2}^{k}$, …, $Z_{2}^{M\times N}$, which is according to the monotonicity of the camera response function, we have the correspondence relationship,

(1) $$Z_{1}^{k}\Leftrightarrow Z_{2}^{k},\;k=1,2,\cdots ,M\times N.$$

Therefore, for photometric-variation images, the identical binary image can be obtained by binarizing any percentile of the ordering pixels.

### 2.2. The Ordinal Measures

The MTB, Local Binary Pattern (LBP) [29], and Local Ternary Patterns (LTP) [30] are often used to represent the illumination invariance of image. Wu [16] proposed the MTB because it can obtain the best features for different illumination images. The mathematical expression is shown by:

(2) $$F_{MTB}\left(u\right)=\left\{\begin{array}{c} 1,\;if\;Z\left(u\right)>z_{med}\\ 0,\;otherwise \end{array}\right.,$$

where the *u* is a point in the image *Z*, the $Z\left(u\right)$ is intensity value of point *u*, and the $z_{med}$ is the median.

However, Wu [31] pointed out that MTB also has some problems as: (1) the same gray value in the discrete domain has many pixels, so it is impossible to achieve absolute equal segmentation with the median; (2) the conversion is very sensitive to noise, especially for pixels that are close to the median; and (3) this conversion is less accurate in taking extreme values in very dark or high-brightness images (which is close to 0 or 255). In order to solve the problems, the multiple-optimal image binarization method is introduced.

### 2.3. Multiple-Optimal Image Binarization Method

Note that $Z_{1}$ and $Z_{2}$ are the two images of the same scene, and $\Pi_{1}$ and $\Pi_{2}$ are the corresponding cumulative distribution. The optimal percentile ξ($\xi_{1}$,$\xi_{2}$) based on ordinal information to binarize images $Z_{1}$ and $Z_{2}$ are obtained by:

(3) $$\xi_{1},\xi_{2}=\arg\min_{p,q}\left|\Pi_{1}\left(p\right)-\Pi_{2}\left(q\right)\right|,$$

where *p* and *q* are the gray values, the $p,q\in \left[0,\;255\right]$, and the minimum value is 0 when both *p* and *q* equal to 255. To avoid this, and to eliminate the noise appearing in the shadow image, the search range was limited to [50, 250]. In order to further improve the robustness of the method, the introduced multiple binarizations method to obtain a series of new images:

(4) $$B_{1}^{k}\left(u\right)=\left\{\begin{array}{c} 1,\;\;if\;\;Z_{1}\left(u\right)>\xi_{1}^{k}\\ 0,\;\;otherwise \end{array}\right.,\;\;B_{2}^{k}\left(u\right)=\left\{\begin{array}{c} 1,\;\;if\;\;Z_{2}\left(u\right)>\xi_{2}^{k}\\ 0,\;\;otherwise \end{array}\right.,$$

where the $B_{1}^{k}$ and $B_{2}^{k}$ is the k-th binary image. When the *K* is the total energy level of the original image binarization, that is, the illumination change image is binarized by the suboptimal percentile $\xi_{1}^{k},\xi_{2}^{k}\left(k=1,\;2,\;\cdots ,\;K\right)$.

### 2.4. Eliminating Effect of Photometric Variation

Here, ${\hat{Z}}_{1}$ and ${\hat{Z}}_{2}$ are two smooth images with the same scene and different illumination, which can be linked by:

(5) $${\hat{Z}}_{2}\left(u\right)=f_{12}\left({\hat{Z}}_{1}\left(u\right)\right),\;{\hat{Z}}_{1}\left(u\right)=f_{21}\left({\hat{Z}}_{2}\left(u\right)\right),$$

where $f_{12}$ and $f_{21}$ are known as the Intensity Mapping Functions (IMFs) [32]. The $f_{12}\left(f_{21}\right)$ represent the image ${\hat{Z}}_{1}\left({\hat{Z}}_{2}\right)$ to image ${\hat{Z}}_{2}\left({\hat{Z}}_{1}\right)$ mapping strength. IMFs can be calculated by histogram matching as shown by:

(6) $$f_{12}\left(z_{1}\right)=\Pi_{2}^{-1}\left(\Pi_{1}\left(z_{1}\right)\right),\;f_{21}\left(z_{2}\right)=\Pi_{1}^{-1}\left(\Pi_{2}\left(z_{2}\right)\right),$$

where the $z_{1}$ and $z_{2}$ are the intensity value of corresponding image ${\hat{Z}}_{1}$ and ${\hat{Z}}_{2}$. In order to determine whether to use $f_{12}$ or $f_{21}$, a weighting function $\omega \left(z\right)$ is introduced for the pixel value at each pixel point, and its mathematical expression is shown by:

(7) $$\omega \left(z\right)=\left\{\begin{array}{cc} \; & z,\;if\;z<128\\ \; & 255-z,\;otherwise \end{array}\right.,$$

where the *z* is the intensity value of single pixel. However, what we need is to perform intensity mapping on the entire image, so the weight of a single pixel is not enough. Therefore, we need to calculate the cumulative weight of all pixels of image ${\hat{Z}}_{1}\left({\hat{Z}}_{2}\right)$, and the expression is as follows:

(8) $$W\left({\hat{Z}}_{1}\right)=\sum_{u}\omega \left({\hat{Z}}_{1}\left(u\right)\right),\;W\left({\hat{Z}}_{2}\right)=\sum_{u}\omega \left({\hat{Z}}_{2}\left(u\right)\right),$$

where $W\left({\hat{Z}}_{1}\right)$ and $W\left({\hat{Z}}_{2}\right)$ are the cumulative weight of the image ${\hat{Z}}_{1}$ and ${\hat{Z}}_{2}$. Further, we determine whether to transform the image by comparing the cumulative weight of the two images. Finally, normalize the input image. The result is as follows

(9) $$\begin{array}{cc} \; & {\bar{Z}}_{1}=\left\{\begin{array}{c} f_{21}\left({\hat{Z}}_{2}\right)\;if\;W\left({\hat{Z}}_{1}\right)<W\left({\hat{Z}}_{2}\right)\\ \;{\hat{Z}}_{1}\;otherwise \end{array}\right.\\ \; & {\bar{Z}}_{2}=\left\{\begin{array}{c} f_{12}\left({\hat{Z}}_{1}\right)\;if\;W\left({\hat{Z}}_{2}\right)<W\left({\hat{Z}}_{1}\right)\\ \;{\hat{Z}}_{2}\;otherwise \end{array}\right. \end{array}.$$

The key of this section is to use a reliable (less saturated) image to map the intensity, which can significantly reduce the effect of image saturation, eliminate the effect of large-photometric-variation on the image, improve detection environment, and reduce the difficulty of feature point detection.

## 3. Feature Point Detection Based on Neighborhood Information

Detection method based on feature point neighborhood information can be further divided into two types, namely the detection method based on the number of feature point neighborhood connections and the detection method based on the location of feature point neighborhood connections. The former has been introduced in Reference [16], we will focus on introducing the latter in this paper.

### 3.1. Classification Based on Neighborhood Connectivity Location

Different from the classification method based on the number of neighborhood connections, the classification method based on the location of neighborhood connections not only contains the number information of neighbors but also contains the location information.

**Figure 1:**
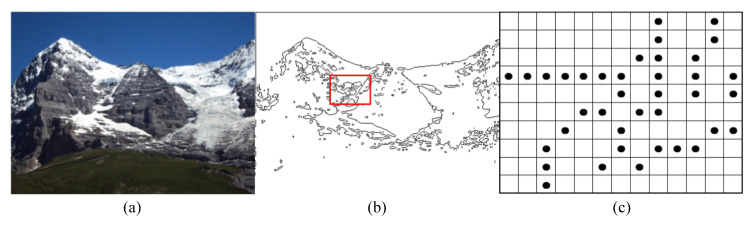
Candidate feature point extraction process. (**a**) Original image. (**b**) Edge feature. (**c**) Local candidate feature points map.

**Figure 2:**
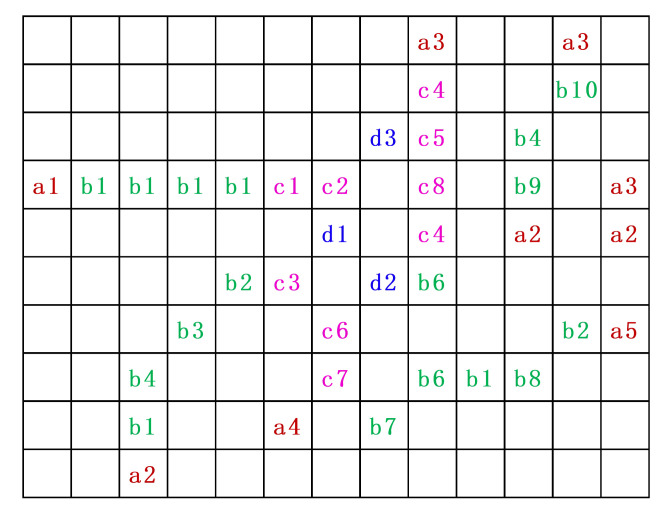
Feature point neighborhood connectivity information.

Figure 1c is a local candidate feature points map of Figure 1a, and the diagram of feature point neighborhood connectivity information is shown in Figure 2.

**Figure 3:**
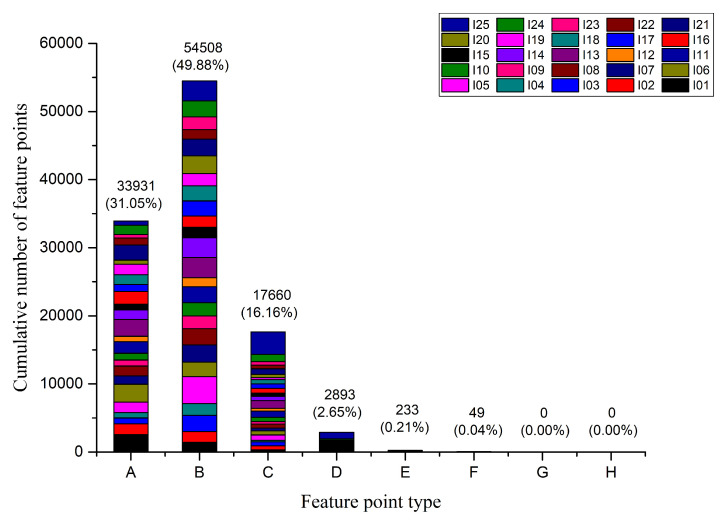
Feature point type statistics. A to H are Endpoint, Corner, Junction, Intersection, Five-line intersection, Six-line intersection, Seven-line intersection, and Eight-line intersection in turn. I01 to I25 are the numbers of 25 images in TID2008.

Each combination of letters and numbers in Figure 2 represents a candidate feature point. Different letters indicate that the number of neighboring connections is different. The letters are the same and the numbers are different, indicating that the neighboring pixels are different connected location. Furthermore, the feature point neighborhood contains up to eight pixels, that is, there can be up to eight directions. Therefore, based on the number of neighboring feature points we can divide feature point into eight types: Endpoint, Corner, Junction, Intersection, Five-line intersection, Six-line intersection, Seven-line intersection, and Eight-line intersection. Here, we count the number and proportion of different types of feature points in the image. The experimental material was derived from the TID2008 dataset. The statistical results are shown in Figure 3.

The experimental results indicate: (1) Corner account for the highest proportion, close to 50%. Followed by Endpoint and Junction; (2) the first four types of feature points account for more than 99%; and (3) the latter four types of feature points account for a very small proportion and can be ignored. Therefore, feature detection only needs to detect the first four types of feature points.

**Figure 4:**
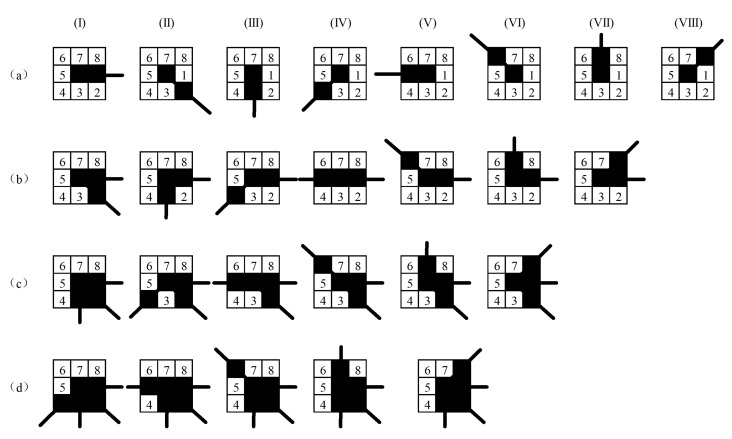
Classification method based on the connection position of feature point neighborhood. (**a**) Endpoint. (**b**) Corner. (**c**) Junction. (**d**) Intersection. The I to VIII are classifications based on connected positions.

In order to further reduce the time spent on matching and improve the matching accuracy, we introduced the location information of the neighborhood, and proposed a feature point classification method based on the connection location of the neighborhood, as shown in Figure 4. It should be particularly noted that the proposed method divides the feature points into 250 types, and it is neither realistic nor necessary to list them all in the paper. Therefore, Figure 4 only shows a part of them for visual analysis.

1. Endpoint
  Different connection positions of neighboring pixels constitute different types of Endpoint. One pixel is arbitrarily connected in the 8 neighborhoods of the feature point to form an Endpoint. Therefore, the Endpoints can be divided into 8 types. The Endpoint type is shown in Figure 4a.
2. Corner
  The feature point is connected with two different pixels in the 8 neighborhoods to form a Corner. Take the I-type Endpoint in Figure 4a as an example, where the Endpoint itself occupies a pixel position, and another pixel is randomly selected from the remaining seven positions to form a Corner. According to the position of the second pixel, the feature points form a new type. Note that, when two neighboring pixels form a straight line with the feature points, as shown in Figure 4b type IV, it is no longer a Corner and needs to be excluded. The Corner can be divided into 24 types.
3. Junction
  Based on the I-type Corner in Figure 4b, the connected pixel is added to the remaining neighborhood position to form a third type of feature point, which is named Junction. Figure 4c shows a Junction that is derived from the Corner of I-type. The Junction can be divided into 56 types.
4. Intersection
  The Intersection is generated based on the Junction. Figure 4d shows several types of Intersection derived from the Junction of I-type. The Intersection can be divided into 70 types. Figure 3 shows that, when the number of connected neighbors of feature points is greater than 4, the probability of occurrence is small, which is not enough to affect the matching result, so it is not considered.

### 3.2. Feature Point Detection

For the photometric-variation image, multiple-optimal image binarization method is used to obtain multiple binarization images. For each binarization image, assuming that $B_{1}$ and $B_{2}$ are the optimal binarization image that is obtained by the optimal percentile $\xi \left(\xi_{1},\;\xi_{2}\right)$. The image target boundary is obtained as follows:

(10) $$P_{j}=B_{j}-\left(B_{j}\Theta \Omega \right),$$

where the $j\in \left\{1,2\right\}$, the Ω is a square structural unit having a width of 3 pixels, and Θ is a corrosion operation.

For the image $P_{j}$ containing the feature points, the image feature point $F_{j}\left(u\right)$ is derived from the number of *k* pixels connected to *u* in the image $P_{j}$, and the mathematical expression of the $F_{j}\left(u\right)$ is shown by:

(11) $$F_{j}\left(u\right)=\sum_{k\in \Theta \left(u\right)}P_{j}\left(k\right),$$

where $\Theta \left(u\right)$ is the 8-connected neighborhood of feature point *u*, and $F_{j}\left(u\right)$ is the number of neighbors of feature point *u* in the j-th image, $F_{j}\left(u\right)\in \left\{1,2,3,4,5,6,7,8\right\}$. When $F_{j}\left(u\right)$ = 1, it means that the feature point is the Endpoint, $F_{j}\left(u\right)\;=\;2$, the feature point is the Corner. Equation (11) is the mathematical expression of the classification method based on the number of connected neighbors.

The detection method based on the connected position of the feature point neighborhood not only needs to obtain the number of connected pixels in the neighborhood around the feature point but also acquires the connected position. The mathematical expression of the proposed method is as follows:

(12) $$F_{j}^{i}\left(u_{k}\right)=\sum_{k\in \Theta \left(u_{k}\right)}P_{j}\left(k\right),$$

where $u_{k}$ represents the specific position of the *k* pixel relative to the feature point *u*, *i* represents the number of connected neighbors of the feature point, *j* represents the corresponding image, and $\Theta \left(u\right)$ is the 8-connected neighborhood of the pixel *u*.

There is the following equivalent relationship between the feature points and their mathematical expressions in the proposed method,

(13) $$F_{j}^{1}\left(u_{1}\right)\Leftrightarrow \left(\begin{array}{ccc} 0 & 0 & 0\\ 0 & u & 1\\ 0 & 0 & 0 \end{array}\right),$$

where $F_{j}^{1}\left(u_{1}\right)$ indicates that the point is an Endpoint, and is the type-I Endpoint.

## 4. Matching Performance Analysis

**Figure 5:**
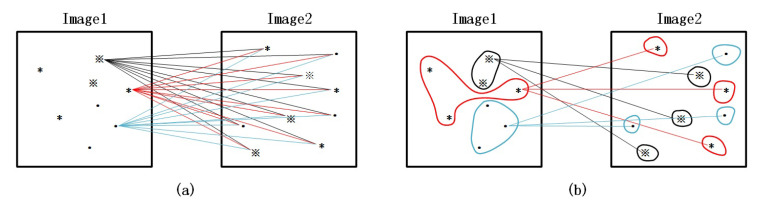
Two matching method. (**a**) General matching method. (**b**) Classification matching method.

Feature point matching is the process of detecting and extracting feature points from the image, and then finding the closest corresponding point according to a preset measurement criterion. Figure 5 shows two different matching ideas. Figure 5a shows the general feature point matching, and Figure 5b shows the classification matching of feature point.

The key to the classification matching is to perform the matching process in a subset of the corresponding classification. The classification matching based on the number of connected neighbors can be shown:

(14) $$F_{1}\left(u\right)\Leftrightarrow F_{2}\left(u\right).$$

The classification matching based on the connected position of the feature point neighborhood can be shown:

(15) $$F_{1}^{i}\left(u_{k}\right)\Leftrightarrow F_{2}^{i}\left(u_{k}\right),$$

where the $u_{k}$ is a subset of the *u*. The number of feature points in $u_{k}$ is less than that in *u*.

### 4.1. Matching Time Estimation

Element $z_{ij}$ in *W* represents a measure of similarity between feature point $x_{i}$ and $y_{j}$, the kernel function K:X×Y→R is used to define these elements as inner products in an inner product space, and the mathematical expression is as shown by:

(16) $$z_{ij}=K\left(x_{i},y_{j}\right)=\langle \phi \left(x_{i}\right),\phi \left(y_{j}\right)\rangle ,$$

and the time cost of two matching point pairs,

(17) $$Z=\left(\begin{array}{ccc} z_{11} & \ldots & z_{1n}\\ \vdots & \ddots & \vdots \\ z_{m1} & \cdots & z_{mn} \end{array}\right)=\left(\begin{array}{ccc} K\left(x_{1},y_{1}\right) & \ldots & K\left(x_{1},y_{n}\right)\\ \vdots & \ddots & \vdots \\ K\left(x_{m},y_{1}\right) & \cdots & K\left(x_{m},y_{n}\right) \end{array}\right).$$

In general, given an appropriate kernel function *K*, Mercer’s theorem [33] ensures that there is an inline function $\phi \left(\cdot \right)$; in this paper, the time cost of two matching point pairs is estimated by Equation (17).

### 4.2. Matching Time Comparison

For the traditional feature point matching method, such as SIFT, the time consumed by the matching is equal to the inner product of each feature point $x_{i}$ and $y_{j}$, and time consumption are obtained by

(18) $$T\mathrm{ime}\left(Z\right)=\sum_{i=1}^{m}\sum_{j=1}^{n}time\left(K\left(x_{i},y_{j}\right)\right).$$

Based on the detection method of the number of neighborhood of feature points, the feature point set *X* of image $Z_{1}$ is segmented into eight feature point sets: $X_{1},\;X_{2},\;\cdots ,\;X_{7},\;X_{8}$. The feature point matching time overhead can be described by

(19) $$T\mathrm{ime}\left(Z^{\prime }\right)=\sum_{i=1}^{8}time\left(K\left(X_{i},Y_{i}\right)\right).$$

Further, in the feature point detection method based on the connected position of feature points, the feature point set is further refined and divided into endpoint $X_{1}^{i}$, corner $X_{2}^{j}$, junction $X_{3}^{k}$, intersection $X_{4}^{h}$, etc. The following relationship exists after the division:

(20) $$X_{1}^{i}\in X_{1},\;X_{2}^{j}\in X_{2},\;X_{3}^{k}\in X_{3},\;X_{4}^{h}\in X_{4}.$$

The time required for feature point matching in the feature point detection method based on the feature point neighborhood connected position is expressed by:

(21) $$T\mathrm{ime}\left(Z^{\prime \prime }\right)=\underset{s.t.\;p\in \left\{1,2,3,4\right\},\;q=C_{8}^{p}}{\sum_{j=1}^{p}\sum_{i=1}^{q}time\left(K\left(x_{p}^{q},y_{p}^{q}\right)\right)}\;.$$

For example, $K\left(x_{1}^{i},y_{1}^{i}\right)$ is the kernel function of the set of points formed by the type *i* endpoints in the endpoint, and $time\left(K\left(x_{1}^{i},y_{1}^{i}\right)\right)$ is the time it takes for the type *i* endpoint to match.

According to Equations (18), (19) and (21), it can be judged that the time overhead of feature point matching has the following relationship:

(22) $$T\mathrm{ime}\left(Z\right)>T\mathrm{ime}\left(Z^{\prime }\right)>T\mathrm{ime}\left(Z^{\prime \prime }\right).$$

## 5. Experimental Results

In this section, we selected images with different illumination as experimental materials for feature point detection and matching. Some of these images are obtained by changing the exposure settings, and the others are captured at different time periods, such as morning and afternoon, daytime, and night. The experimental materials include indoor scenes, outdoor scenes, close-up, and long-distance scenes.

The comparison methods used in this paper include two types, which are feature-based and learning-based detection methods. Feature-based methods include Harris [11], MinEigen [34], SIFT [12], SURF [13], IRFET_Harris [8], FAST [35], ORB [36], A-KAZE [37], and Wu [16]. In this section, unless otherwise specified, the Wu’s methods [16] are all denoted as Wu. The learning-based methods select LIFT [20], SuperPoint [22], and LF-Net [23]. The relevant parameters of the feature-based method all follow the parameters in the published paper, and the learning-based detection method uses the pre-trained model published by the author of the paper on github. The original setting number of keypoints in the LIFT and LF-Net pre-training models is small (LIFT is 1000 and LF-Net is 500), which seriously affects the fairness of the experimental result. In order to avoid the unfairness, we uniformly set the maximum number of keypoints in the pre-training model to a very large value to ensure that the most feature points can be detected.

We use several common feature detector evaluation indicators including the number of feature points, the number of matching points, and the repeatibility rate to evaluate the performance of the proposed method. Repeatibility rate is a key evaluation indicator, with various definitions, among which the definition of [38] is widely used, and the expression is as follows:

(23) $$r\left(d\right)=\frac{\left\{\left({\tilde{x}}_{i},{\tilde{x}}_{j}\right)\left|dist\left(H_{ij}{\tilde{x}}_{i},{\tilde{x}}_{j}\right)\right.<d\right\}}{\min\left(n_{i},n_{j}\right)},$$

where $\left({\tilde{x}}_{i},{\tilde{x}}_{j}\right)$ is a pair of matching feature points, $dist(H_{ij}{\tilde{x}}_{i},{\tilde{x}}_{j})$ is the distance between the pair of matching feature points, and the $H_{ij}$ is a homography matrix, used to transform point ${\tilde{x}}_{i}$ in one image to another image.

### 5.1. Different Exposure Value

**Figure 6:**
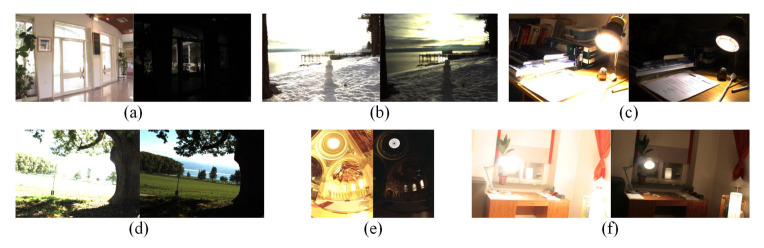
Experiment material of different exposure value. (**a**) Belgium. (**b**) SnowMan. (**c**) CadikDesk. (**d**) BigTree. (**e**) Memorial. (**f**) WindowSeries.The left image of each group is overexposed, and the right image is underexposed.

Figure 6 contains six groups of experimental materials with different exposure value. Each group of materials consists of two images of the same scene. The left image is overexposed, and the right image is underexposed.

**Figure 7:**
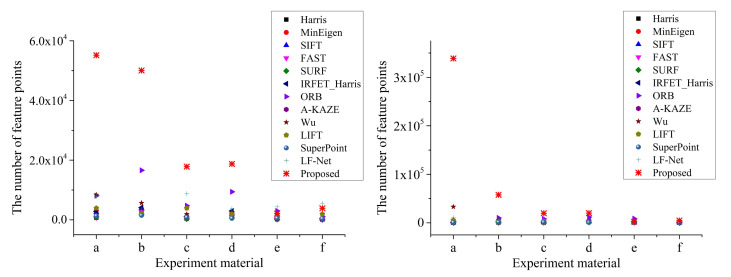
Number of feature points. On the left are the feature points extracted from the overexposed images, and on the right are the feature points extracted from the underexposed images. The X-axis serial number corresponds to experimental materials in Figure 6.

**Figure 8:**
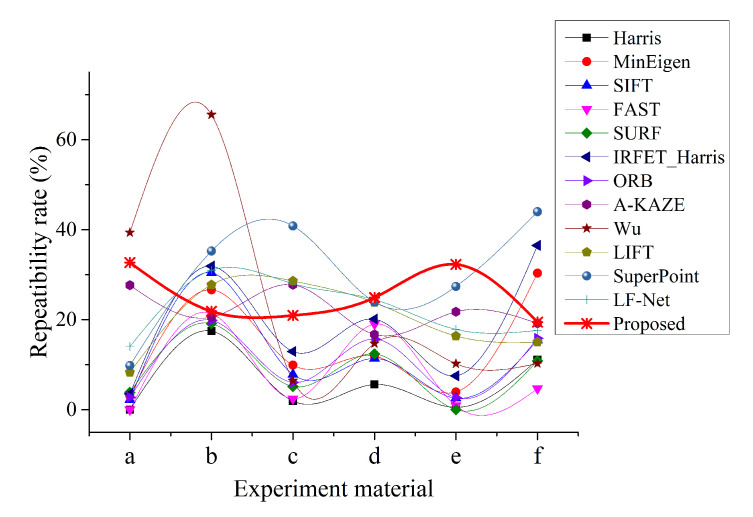
Repeatibility rate change curve of feature detector.

The number of feature points is shown in Figure 7. The left experimental result corresponds to the overexposed images in Figure 6, and the right experimental result corresponds to the underexposed images. The number of matching points is shown in Table 1. The repeatibility rate evaluation value based on Equation (23) is shown in Figure 8.

The number of feature points is one of the most important performance evaluation indicators for feature detectors. Figure 7 indicates that our method can extract a large number of significant feature points from two images with large-photometric-variation. In most cases, our method can obtain the most feature points, and, in the remaining few experimental results, although the number of feature points extracted by the proposed method is not the most, it can still be guaranteed to be at the upper-middle level. In addition, ORB and LF-Net also show excellent performance in terms of the number of feature points extracted, sometimes even more than the proposed algorithm.

In addition, the number of matching points is another important evaluation indicator. In this article, we use the number of theoretical matching feature points and the actual number of matching feature points for algorithm evaluation. The calculation method of theoretical matching feature points is as follows. (1) First, extract feature points from underexposed images and overexposed images. (2) Secondly, the feature points in the overexposed image are transformed into the underexposed image through the homography matrix (since the scene is the same, the homography matrix here can be simplified to a unit matrix). (3) Finally, check whether there is a feature point at the corresponding position of the underexposed image. If it exists, we consider this pair of feature points as theoretical matching feature points. Table 1 shows the number of theoretical matching feature points. The experimental result is obtained by Equation (23).

In the first four groups of experimental results, the proposed method has obvious advantages. The number of matching points is several to several tens of times that of other detection methods. In the last two groups of experiments results, the proposed method is equivalent to LF-Net algorithm.

LF-Net shows very good performance in the matching experiment of large-photometric-variation, which is only slightly inferior to the proposed method; LIFT and A-KAZE are inferior to the former, but they perform well in terms of the number of matching points and matching stability; ORB and Wu can obtain a large number of matching feature points under certain scenes and illumination conditions, but their performance is not stable enough. In addition, Harris, FAST, and SURF perform extremely poorly under large-photometric-variation, and sometimes even a pair of matching points cannot be obtained.

In addition to the number of feature points and matching points, the repeatibility rate is also a commonly used evaluation indicator. It intuitively reflects the proportion of matching feature points in the extracted feature points and is used to characterize the availability and repeatibility of the feature points extracted by the feature detector. The repeatibility rate is shown in Figure 8.

Figure 8 shows that the repeatibility rate of the proposed method is not the highest in most cases, but it is the most stable, basically around 30%, with a small fluctuation range of 20% to 40%. On the contrary, the repeatability rate of other methods fluctuates greatly. For example, Wu’s method has a repeatibility rate of 60% at the highest and close to 0 at the lowest. The repeatibility rate of SuperPoint exceeds 40% at the highest and about 10% at the lowest. Combining Figure 7 and Figure 8 and Table 1, we find that the proposed method can extract the most feature points and obtain the most matching feature points, while the repeatibility rate changes the most stable. Therefore, we believe that the proposed method has the best illumination robustness.

However, this is not enough because we also need to verify whether the matching points can indeed be used for feature point matching in the real environment. The calculation method of actual matching feature points is as follows. First, extract the feature points from the two images; then, calculate the descriptor for each extracted feature point; finally, select the appropriate matching algorithm for feature point matching and calculate the actual number of matching feature points. Table 2 shows the actual number of matching points (the same descriptor and matching method were used in the previous period).

There is a certain deviation between the data in Table 1 and Table 2. However, the proposed method still obtains the most matching feature points in most cases. Although the actual number of matching feature points in the other two groups is not the most, it performs well in the same group of experiments. In addition, although LF-Net performance is not as good as the proposed method in terms of the matching points number of theoretical calculations, the experimental results of “CadikDesk” and “Memorial” have exceeded the proposed method in actual matching experiments. At the same time, the experimental results of “BigTree” and “WindowSeries” are very close to the proposed method, which indicate that LF-Net also has excellent illumination robustness. In addition to LF-Net, SuperPoint and LIFT also surpass most feature-based detection methods (except the proposed methods) in the actual feature points matching experiment.

In order to further verify the previous experimental results, we give the alignment and overlay images of different experimental groups, as shown in Table 3.

The experimental results in Table 3 indicate that the alignment based on Harris and FAST is the worst; LIFT, SuperPoint, LF-Net, and the proposed method perform best in the image alignment experiments, and all can achieve correct image alignment. “Belgium” and “Memorial” have the largest illumination differences, so most feature detectors fail in these two experiments. “SnowMan”, “CadikDesk”, and “BigTree” are relatively difficult, so most detectors can extract enough matching feature points and perform correct alignment. The alignment results in Table 3 can well prove the previous experimental results.

### 5.2. Different Capture Time

**Figure 9:**
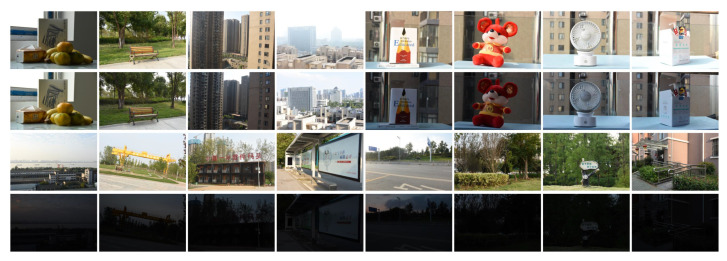
Images of different illumination. The first and second rows correspond to the same scene, the capture time of the first row is in the morning, and the capture time of the second row is in the afternoon. The third and fourth rows correspond to the same scene, the third row of images were captured during the daytime, and the fourth row was captured at night.

When the camera settings and pose are fixed and only the capture time is different, a series of images with different illumination directions or intensities can be obtained, as shown in Figure 9. The first and second rows correspond to the same scene, the capture time of the first row is in the morning, and the capture time of the second row is in the afternoon. Therefore, we collectively refer to the first two rows as Morning-Afternoon dataset. The third and fourth rows correspond to the same scene, the third row of images were captured during the daytime, and the fourth row was captured at night. We call the last two rows Daytime-Night dataset. From left to right, the first column is named Scene_1, the second column is named Scene_2, and so on.

We extracted the feature points of each pair of images in the Morning-Afternoon dataset and shown them in Table 4 and Table 5.

The images in the Morning-Afternoon dataset show different states in different areas due to the different directions of sunlight. The originally bright area may become darker, and the originally darker area may become brighter. This makes it more difficult to match the feature points of the image.

Compared with other methods, the ORB, Wu’s method, LIFT, LF-Net, and the proposed method can extract more feature points when the illumination in different areas of the same scene changes significantly. Further, we count the number of theoretical matching points, and the statistical results are shown in Table 6.

The experimental results in Table 6 indicate that the proposed method can still obtain the most matching feature points when the image illumination direction changes. However, the situation reflected by Scence_3 cannot be ignored. When the light-dark area is completely reversed, the proposed method may not work well. In addition, ORB, Wu’s method, and LF-Net can also theoretically extract many matching feature points.

The number of theoretical matching feature points is obtained by Equation (23), which does not consider feature descriptors and matching methods, so interference caused by algorithm compatibility can be eliminated. However, the number of theoretical matching points is extremely dependent on the control accuracy of the camera pose during the image capture process. Therefore, in addition to counting the number of theoretical matching points, we also need to further examine the actual number of matching feature points, and comprehensively consider the two to ensure the credibility of the result. The actual number of matching feature points is shown in Table 7 (the same descriptor and matching method were used in the previous period).

In the 8 groups of experiments, 5 groups of proposed methods obtained the most matching feature points, and the other two groups ranked second, and the result of one group was poor (Scene_3). LF-Net followed closely behind.

When the illumination direction changes, Wu’s method, LIFT, LF-Net, and the proposed method can perform well in terms of the number of feature points and the number of matching points. In addition to considering the change of illumination direction, we also further consider the change of illumination intensity, as shown in the Daytime-Night dataset in Figure 9.

The illumination intensity of the two images in the Daytime-Night dataset is very different, so it is more difficult to use feature detection methods to extract feature points from low-illuminance images and match them with other images. The number of feature points extracted by different feature detection methods from the Daytime-Night dataset is shown in Table 8 and Table 9.

The images in Table 8 are captured during the daytime, so all detection methods can extract enough feature points. The images in Table 9 are different. Because it is captured after the sun sets, the illumination is very poor, so the difficulty of extracting feature points is greatly increased. However, there are also some detection methods that can extract feature points from low-illumination images, such as Wu’s method, LIFT, SuperPoint, LF-Net, and the proposed method. Further, we conducted statistics on the number of theoretical matching points and obtained the experimental results shown in Table 10.

Similarly, only Wu’s method, LIFT, SuperPoint, LF-Net, and the proposed method can obtain better matching results. The matching points of the proposed method is much higher than other methods, which indicates that the proposed method has the potential to obtain the most matching points under low illumination. In addition, the statistical results of actual matching points are shown in Table 11.

The actual matching point statistics indicate that the proposed method can still obtain the most matching feature points, but the advantages are reduced compared to Table 10. For example, the actual matching feature points of the proposed methods in Scene_5 and Scene_7 are very close to LF-Net. In addition, the feature points extracted by LF-Net have obvious performance advantages in the actual matching process, far exceeding other algorithms, followed by Wu’s method, SuperPoint and LIFT detection methods.

Through the analysis of the experimental results of the day-night dataset, it is found that, except for the methods of IRFET_Harris and Wu, other feature-based detection methods are difficult to extract enough feature points for matching. In contrast, learning-based methods have good phenotypes in terms of the number of feature points and the number of matching points, especially LF-Net, which has excellent illumination robustness. However, our proposed method surpasses LF-Net in all performance evaluation indicators. Furthermore, through analysis of the number of theoretical matching points and actual matching points, it is found that, due to the limitation of feature description and matching methods, many feature points cannot be matched correctly.

## 6. Discussion

This paper focuses on the illumination robustness of feature detection methods. In order to make the results more convincing, we used three types of data sets with different exposure values, different light directions, and different light intensities. For each data set, the proposed method and the other twelve feature detection methods are used for feature detection, extraction, and matching. Finally, the number of feature points and the number of matching points is used as evaluation indicators.

The experimental results of the three data sets are generally consistent, but due to the characteristics of the data sets themselves, the experimental results also have some differences in some details. In datasets with different exposure values, in addition to Wu, LIFT, SuperPoint, LF-Net, and the proposed method, the experimental results of other methods are not good. The reason is that the two images contained in each pair of experimental materials are underexposed images and overexposed images, respectively. Wu and the proposed method use multi-optimal image binarization to resist this large photometric variation. The other three groups of learning-based methods may have considered large photometric variation during the training process.

In the experiment where the illumination direction changes, most detection methods can extract enough feature points, which indicates that the change of the illumination direction has little effect on the detection method.

The last data set contains two images with different light intensities. Images captured during the daytime can extract enough feature points, while images captured at night have two extremes when extracting feature points. Some methods, including the proposed method and three learning-based methods, can still extract feature points equivalent to those during the daytime, but other methods cannot detect feature points at all. By comparing and analyzing the experimental results of the three data sets, we can conclude that the proposed method has the best illumination robustness.

## 7. Conclusions

In this paper, we proposed a novel feature point detector based on neighborhood connected information, which classifies and detects feature points based on the number and location information of the eight neighborhoods of the pixels to be detected. The proposed detector is proved to have better detection ability than other detectors in the case of under-exposure and over-exposure. This indicates that our method has the best illumination robustness. At the same time, it is also superior to other methods in terms of matching accuracy and matching time consumption. The experimental results also verify the above conclusion.

The proposed method also has some disadvantages. For matching accuracy, our method abandons geometric invariance. In other words, this method is not suitable for feature point detection under rotation or affine transformation. In the future, if the homography matrix of geometric transformation can be calculated, the proposed method can be broadened to geometric invariance of feature detection.

## Figures and Tables

**Table 1:** Number of matching points obtained through theoretical calculation. The bold font indicates that the data obtained the best results in the same group of experiments.

Method∖Material	Belgium	SnowMan	CadikDesk	BigTree	Memorial	WindowSeries
Harris	0	74	7	48	1	11
MinEigen	24	822	111	297	17	150
SIFT	12	722	67	226	12	50
FAST	0	145	11	293	4	3
SURF	9	110	15	79	0	14
IRFET_Harris	31	1059	140	604	43	146
ORB	76	1978	287	1473	80	180
A-KAZE	16	209	24	217	4	27
Wu	3297	3698	62	281	37	46
LIFT	324	618	881	401	313	289
SuperPoint	55	425	334	121	147	165
LF-Net	1102	1116	2059	776	672	737
Proposed	**18,043**	**10,935**	**3729**	**4666**	**679**	**754**

**Table 2:** Actual number of matching points. The same descriptor and matching method were used in the previous period. The bold font indicates that the data obtained the best results in the same group of experiments.

Method∖Material	Belgium	SnowMan	CadikDesk	BigTree	Memorial	WindowSeries
Harris	5	30	6	6	2	10
MinEigen	7	579	70	59	6	101
SIFT	5	229	18	17	1	25
FAST	0	56	9	32	0	7
SURF	7	55	16	15	0	22
IRFET_Harris	4	767	79	114	6	79
ORB	2	624	89	61	2	39
A-KAZE	3	59	24	26	1	20
Wu	101	1851	19	28	3	25
LIFT	39	555	774	316	87	267
SuperPoint	16	414	273	63	40	141
LF-Net	61	927	**1312**	616	**137**	412
Proposed	**1308 **	**17,434**	457	**674**	49	**503**

**Table 3:** Actual number of matching points. Blank means that the feature detector cannot find enough feature points on the corresponding experimental material for image registration. The red frame area indicates that the alignment and overlay results are incorrect due to registration errors.

Method∖Material	Belgium	SnowMan	CadikDesk	BigTree	Memorial	WindowSeries
Harris	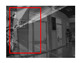	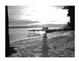	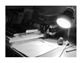	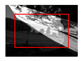		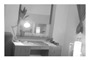
MinEigen	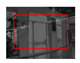	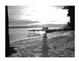	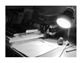	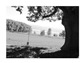	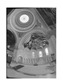	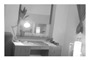
SIFT	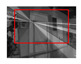	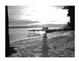	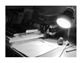	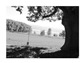		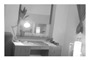
FAST		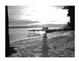	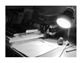	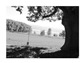		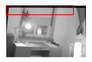
SURF	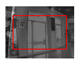	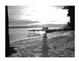	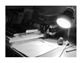	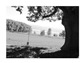		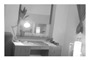
IRFET_Harris	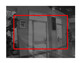	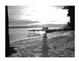	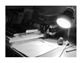	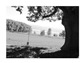	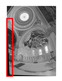	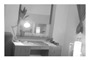
ORB		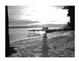	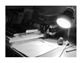	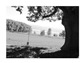		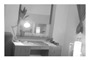
A-KAZE		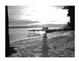	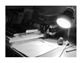	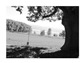		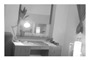
Wu	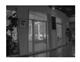	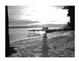	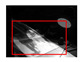	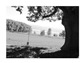		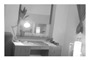
LIFT	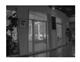	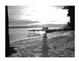	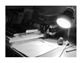	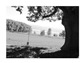	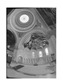	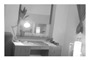
SuperPoint	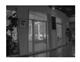	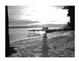	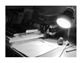	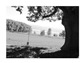	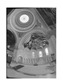	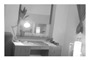
LF-Net	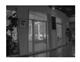	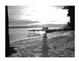	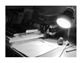	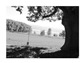	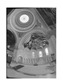	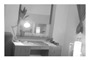
Proposed	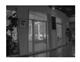	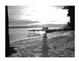	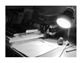	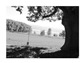	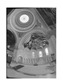	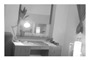

**Table 4:** Feature points obtained from the image captured in the morning. The bold font indicates that the data obtained the best results in the same group of experiments.

Method∖Material	Scene_1	Scene_2	Scene_3	Scene_4	Scene_5	Scene_6	Scene_7	Scene_8
Harris	112	3370	1388	734	323	479	107	299
MinEigen	1223	14,248	5034	3413	586	1864	470	1170
SIFT	948	7260	3221	1540	797	1253	632	1021
FAST	96	5669	1406	473	537	362	136	401
SURF	263	2567	1201	690	659	374	410	492
IRFET_Harris	1252	17,632	5002	2779	629	1615	481	1056
ORB	3389	**55,660**	**15,199**	6771	3615	5465	2592	4476
A-KAZE	547	3535	1800	984	1146	872	930	873
Wu	2179	13,027	4120	13,511	9081	6409	4974	7864
LIFT	3643	5939	5561	5365	3400	4407	3429	4091
SuperPoint	856	1320	4118	1809	804	592	709	912
LF-Net	**9747**	11,060	10,263	10,341	6982	7808	6574	7479
Proposed	7144	22,576	4937	**23,076**	**24,321**	**15,517**	**13,657**	**19,741**

**Table 5:** Feature points obtained from the image captured in the afternoon. The bold font indicates that the data obtained the best results in the same group of experiments.

Method∖Material	Scene_1	Scene_2	Scene_3	Scene_4	Scene_5	Scene_6	Scene_7	Scene_8
Harris	470	2173	744	2775	381	243	241	260
MinEigen	1514	9049	3863	7746	698	757	820	1550
SIFT	1282	5432	2586	5237	586	703	614	885
FAST	323	5289	1042	2697	236	273	169	185
SURF	404	1727	938	2091	257	138	366	387
IRFET_Harris	1581	11,797	4338	8491	638	852	650	1001
ORB	5271	**37,311**	**12,852**	28,714	2419	2810	2881	3111
A-KAZE	780	2570	1350	3302	425	338	733	645
Wu	2290	11,516	5330	20,062	11,444	6676	6807	8781
LIFT	3641	5629	5074	5539	3595	4365	3475	4324
SuperPoint	895	1372	3665	2363	544	538	746	905
LF-Net	**9041**	10,999	10,983	9966	8578	8999	7482	8171
Proposed	7529	22,880	11,293	**30,355**	**25,270**	**16,643**	**16,642**	**19,129**

**Table 6:** Number of theoretical matching feature points (Morning-Afternoon dataset). The bold font indicates that the data obtained the best results in the same group of experiments.

Method∖Material	Scene_1	Scene_2	Scene_3	Scene_4	Scene_5	Scene_6	Scene_7	Scene_8
Harris	20	133	101	67	45	25	4	114
MinEigen	243	1070	1508	405	73	78	59	660
SIFT	114	375	459	140	30	48	20	227
FAST	19	629	123	51	61	23	11	79
SURF	39	60	81	50	20	9	9	107
IRFET_Harris	255	1836	1748	360	92	158	54	620
ORB	771	6116	**2540**	879	505	613	331	1079
A-KAZE	79	114	146	95	40	48	40	204
Wu	353	2282	287	2948	1097	1002	654	1324
LIFT	183	250	1149	366	111	389	179	782
SuperPoint	108	19	1676	212	36	31	73	422
LF-Net	983	1143	2138	1031	564	999	556	1599
Proposed	**3446**	**10,719**	853	**10,535**	**9195**	**7032**	**5644**	**9147**

**Table 7:** Actual number of matching points (Morning-Afternoon dataset). The bold font indicates that the data obtained the best results in the same group of experiments.

Method∖Material	Scene_1	Scene_2	Scene_3	Scene_4	Scene_5	Scene_6	Scene_7	Scene_8
Harris	71	123	12	37	187	24	14	67
MinEigen	827	384	100	112	276	110	37	342
SIFT	301	112	18	35	67	58	20	43
FAST	63	294	13	20	151	26	15	44
SURF	145	107	11	34	109	30	24	73
IRFET_Harris	857	**594**	106	109	293	114	42	330
ORB	710	375	54	54	339	88	46	321
A-KAZE	272	153	10	45	172	73	50	95
Wu	306	206	30	233	373	290	173	458
LIFT	846	223	190	90	282	314	172	489
SuperPoint	525	92	177	77	192	107	81	238
LF-Net	**1889**	381	**273**	125	495	499	326	606
Proposed	1203	424	19	**260**	**1785**	**1079**	**573**	**1797**

**Table 8:** Feature points obtained from the image captured in the daytime (Daytime-Night dataset). The bold font indicates that the data obtained the best results in the same group of experiments.

Method∖Material	Scene_1	Scene_2	Scene_3	Scene_4	Scene_5	Scene_6	Scene_7	Scene_8
Harris	813	2111	5483	1894	2099	7095	1706	1830
MinEigen	2349	6170	11,166	4228	10,600	16,895	8545	8172
SIFT	2257	4304	7703	3341	3492	12,597	6170	5744
FAST	792	2435	7287	2058	2516	13,350	1265	2349
SURF	888	1418	3111	1364	1142	4511	1373	2405
IRFET_Harris	2446	7083	13,777	5021	9396	22,403	8985	9602
ORB	8275	24,801	**52,457**	16,126	**22,129**	**82,342**	**18,076**	**30,391**
A-KAZE	1513	2147	4435	2243	1878	6780	2128	3293
Wu	7124	19,987	19,846	15,590	4370	14,493	2357	5550
LIFT	5301	5288	5152	4644	5701	6442	6433	5070
SuperPoint	1510	1737	2101	1524	1299	2393	1728	3365
LF-Net	8424	10,382	11,403	9617	11,100	13,207	10,006	11,437
Proposed	**14,792**	**41,063**	39,888	**35,138**	10,742	39,636	7980	15,409

**Table 9:** Feature points obtained from the image captured in the night (Daytime-Night dataset). The bold font indicates that the data obtained the best results in the same group of experiments.

Method∖Material	Scene_1	Scene_2	Scene_3	Scene_4	Scene_5	Scene_6	Scene_7	Scene_8
Harris	1	1557	3208	100	492	2532	699	139
MinEigen	1	2940	5936	513	1532	4355	3385	1637
SIFT	0	366	1563	66	401	2201	240	43
FAST	1	0	0	45	31	931	0	1
SURF	0	20	24	7	53	238	41	3
IRFET_Harris	4	2269	5209	222	1217	4396	1091	245
ORB	7	838	4453	118	1726	8944	467	262
A-KAZE	0	18	32	0	86	331	42	6
Wu	8331	19,713	18,357	18,729	5445	13,280	2242	7735
LIFT	4962	5147	5347	4355	5074	6031	6074	4784
SuperPoint	845	1140	1047	806	1057	1335	641	1033
LF-Net	10,386	12,545	11,628	10,375	**10,907**	10,671	**9236**	10,135
Proposed	**16,702**	**39,881**	**35,768**	**33,985**	10,406	**33,886**	7493	**18,187**

**Table 10:** Number of theoretical matching feature points (Daytime-Night dataset). The bold font indicates that the data obtained the best results in the same group of experiments.

Method∖Material	Scene_1	Scene_2	Scene_3	Scene_4	Scene_5	Scene_6	Scene_7	Scene_8
Harris	1	312	515	42	82	1300	185	62
MinEigen	1	819	1377	206	378	2410	836	778
SIFT	0	55	380	15	61	778	40	15
FAST	1	0	0	20	20	854	0	1
SURF	0	0	9	5	15	162	4	1
IRFET_Harris	3	750	1183	120	321	2936	346	136
ORB	1	229	1229	60	510	4715	78	83
A-KAZE	0	2	12	0	18	258	8	4
Wu	1416	5552	6509	3412	1077	4370	646	824
LIFT	640	627	854	925	510	1945	816	1127
SuperPoint	312	368	250	397	160	478	147	456
LF-Net	1332	1828	2478	1637	1490	3696	1185	2099
Proposed	**7277**	**27,196**	**28,536**	**19,147**	**5853**	**28,509**	**6190**	**6976**

**Table 11:** Actual number of matching points (Daytime-Night dataset). The bold font indicates that the data obtained the best results in the same group of experiments.

Method∖Material	Scene_1	Scene_2	Scene_3	Scene_4	Scene_5	Scene_6	Scene_7	Scene_8
Harris	0	314	667	1	101	521	149	19
MinEigen	0	488	1091	159	370	951	619	220
SIFT	0	22	108	1	25	156	13	2
FAST	0	0	0	2	10	322	0	0
SURF	0	8	8	0	17	84	7	0
IRFET_Harris	1	575	1419	26	363	1147	285	43
ORB	0	76	573	1	109	883	19	4
A-KAZE	0	0	10	0	31	113	4	0
Wu	515	760	1303	701	365	847	253	130
LIFT	495	375	616	428	378	926	1449	504
SuperPoint	296	217	263	250	184	284	172	147
LF-Net	750	453	1128	570	576	1561	1581	**692**
Proposed	**1953**	**2627**	**4606**	**1930**	**750**	**5201**	**1707**	617
